# 5-*n*-Butyl-4-[2-(2-ethyl-1-benzothio­phen-3-yl)-3,3,4,4,5,5-hexa­fluoro­cyclo­pent-1-en-1-yl]thio­phene-2-carbaldehyde

**DOI:** 10.1107/S1600536808036453

**Published:** 2008-11-13

**Authors:** Zhen Yang, Congbin Fan, Min Li, Weijun Liu, Gang Liu, Seik Weng Ng

**Affiliations:** a Jiangxi Key Laboratory of Organic Chemistry, Jiangxi Science & Technology Normal University, Nanchang 330013, People’s Republic of China; b Department of Chemistry, University of Malaya, 50603 Kuala Lumpur, Malaysia

## Abstract

The title compound, C_24_H_20_F_6_OS_2_, exhibiting photochromic behaviour, has thienyl and benzothienyl substituents attached to the double-bond C atoms of the envelope-shaped cyclo­pentene ring. The mean planes of aromatic systems form dihedral angles of 43.0 (1) (thien­yl) and 73.8 (1)° (benzothien­yl) with the mean plane of the C—C=C—C portion of the cyclo­pentene ring. This conformation avoids steric hindrance between the *n*-butyl and ethyl substituents. The formyl substituent of the thienyl group, as well as the ethyl substituent of the benzothienyl group, are disordered [occupancies of 0.788 (17):0.212 (17) and 0.64 (5):0.36 (5), respectively].

## Related literature

For the synthesis of the precursors, see: Pu *et al.* (2008 ▶); Ramamurthy & Venkatesan (1987 ▶); Kobatake & Irie (2004 ▶); Zheng *et al.* (2007 ▶). For the crystal structures of other photochromic dithienyl-substituted hexa­fluoro­cyclo­pentenes, see: Congbin *et al.* (2007 ▶); Li *et al.* (2008 ▶); Liu *et al.* (2008 ▶); Pu & Zhou (2007 ▶); Tu *et al.* (2008 ▶); Zhu *et al.* (2007 ▶).
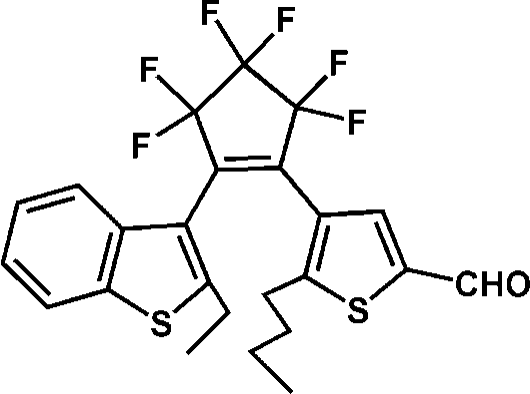


## Experimental

### 

#### Crystal data

C_24_H_20_F_6_OS_2_
*M* *_r_* = 502.52
Triclinic, 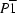

*a* = 10.051 (1) Å
*b* = 11.031 (1) Å
*c* = 12.019 (1) Å
α = 113.126 (1)°
β = 96.882 (1)°
γ = 103.542 (1)°
*V* = 1157.5 (2) Å^3^
*Z* = 2
Mo *K*α radiation
μ = 0.29 mm^−1^
*T* = 296 (2) K
0.43 × 0.43 × 0.43 mm

#### Data collection

Bruker SMART area-detector diffractometer
Absorption correction: none
10086 measured reflections
5195 independent reflections
3669 reflections with *I* > 2σ(*I*)
*R*_int_ = 0.019

#### Refinement

*R*[*F* ^2^ > 2σ(*F* ^2^)] = 0.046
*wR*(*F* ^2^) = 0.151
*S* = 1.03
5195 reflections
318 parameters
30 restraints
H-atom parameters constrained
Δρ_max_ = 0.34 e Å^−3^
Δρ_min_ = −0.25 e Å^−3^

### 

Data collection: *SMART* (Bruker, 1997 ▶); cell refinement: *SAINT* (Bruker, 1997 ▶); data reduction: *SAINT* program(s) used to solve structure: *SHELXS97* (Sheldrick, 2008 ▶); program(s) used to refine structure: *SHELXL97* (Sheldrick, 2008 ▶); molecular graphics: *X-SEED* (Barbour, 2001 ▶); software used to prepare material for publication: *publCIF* (Westrip, 2008 ▶).

## Supplementary Material

Crystal structure: contains datablocks I, global. DOI: 10.1107/S1600536808036453/ya2080sup1.cif

Structure factors: contains datablocks I. DOI: 10.1107/S1600536808036453/ya2080Isup2.hkl

Additional supplementary materials:  crystallographic information; 3D view; checkCIF report

## Figures and Tables

**Fig. 1.**
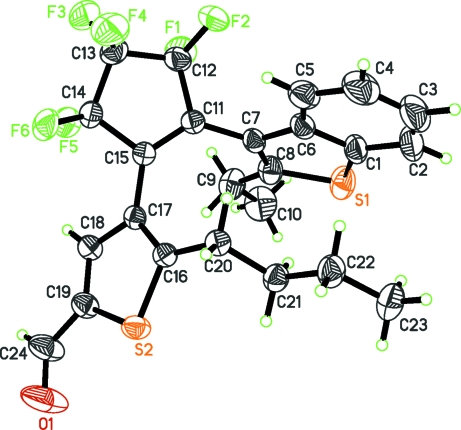
Molecular structure of the title compound and the atom-labelling scheme; thermal displacement ellipsoids are drawn at the 30% probability level. Minor components of the disorder are shown with the dashed bonds; H atoms are omitted.

## supplementary crystallographic information

### Comment

The title compound has thienyl and benzothienyl substituents on the double-bond C atoms of the envelope-shaped cyclopentene ring. The planes of aromatic systems form dihedral angles 43.0 (1)° (thienyl) and 73.8 (1)° (benzothienyl) with the mean plane of the  C4–C11=C15–C16 portion of the cyclpentene ring. Such conformation allows to avoid steric hindrance between the *n*-butyl and ethyl substituents (Fig. 1). The terminal atoms of formyl substituent on the thienyl group as well as the ethyl substituent on the benzothienyl group are disordered over two positions. The intramolecular distance between C3 and C20 is 3.989 (8) Å. This distance indicates that the compound may undergo photochromism in crystalline phase to form the closed ring isomer, as photochromic reactivity was shown to occur when the distance between the potentially reactive C atoms is less than 4.2 Å (Ramamurthy & Venkatesan, 1987; Kobatake & Irie, 2004).

Indeed, crystals of the title compound show photochromism: upon irradiation with 365 nm light, the colourless crystals rapidly turn blue, and the blue crystals turn colourless again upon irradiation with visible light (>510 nm). When dissolved in hexane, the blue compound displays an absorption maximum at 581 nm; the solution of colourless compound has absorption maximum at 257 nm.

### Experimental

2.0 mL (5 mmol) of n-butyllithium was added under stirring in nitrogen atmosphere to 30 mL of THF solution containing 1.46 g (5.0 mmol) of 4-bromo-5-n-butyl-2-(1,3-dioxolane)-thiophene (Zheng *et al.*, 2007) at 195 K. 40 min later, 10 mL of THF solution containing 1.77 g (5.0 mmol) of 1-(2-ethyl-1-benzothien-3-yl)heptafluorocyclopentene (Pu *et al.*, 2008) was added to the reaction mixture and stirring under nitrogen atmosphere at 195 K was continued for two more hours. The reaction mixture was extracted with diethyl ether and evaporated in vacuum. Then the obtained compound was hydrolyzed by *p*-toluenesulfonic acid (0.4 g) in mixture of water (30 ml) and acetone (90 ml). Pyridine (2 ml) was added, and the solution was refluxed for 24 hours and then washed with aqueous sodium bicarbonate. The mixed compound was extracted with diethyl ether and evaporated in vacuum. The crude product was purified by column chromatography on silica, with ethyl acetate and petroleum ether (v/v 1/6) as the eluent, to give 1.58 g (3.15 mmol, 63% yield) of the title compound. Elemental analysis: calc. for C_24_H_20_F_6_OS_2_: C 57.36, H, 4.01%. Found C 57.22, H 3.90%.

### Refinement

All H atoms were positioned geometrically and treated as riding with C—H = 0.97 Å (methylene), 0.96 Å (methyl) or 0.93 Å (aromatic and formyl) with U_iso_(H) = 1.2U_eq_(C) (U_iso_(H) = 1.5U_eq_(C) for methyl H atoms).

The methyl group of the ethyl chain is disordered over two sites C1 and C1'; the the C3–C2 distance was restrained to 1.50±0.01 Å, C2–C1 and C2–C1' distances were restrained to be equal within 0.01 Å, and C3···C1 and C3···C1' restrained to 2.51±0.01 Å. The occupancies of the disorder components refined to a 0.79 (1):0.21 ratio.

The formyl oxygen atom is also disordered over two positions; the C19–O1 and C19-O1' distances were restrained to be equal within 0.01 Å. The occupancies of the O1 and O1' atoms refined to a 0.64 (1):0.36 ratio.

The anisotropic displacement parameters of the disordered atoms were restrained to be nearly isotropic.

### Crystal data

**Table:**

C_24_H_20_F_6_OS_2_	*Z* = 2
*M**_r_* = 502.52	*F*_000_ = 516
Triclinic, *P*1	*D*_x_ = 1.442 Mg m^−^^3^
Hall symbol: -P 1	Mo *K*α radiation λ = 0.71073 Å
*a* = 10.051 (1) Å	Cell parameters from 4259 reflections
*b* = 11.031 (1) Å	θ = 2.5–28.2º
*c* = 12.019 (1) Å	µ = 0.29 mm^−^^1^
α = 113.126 (1)º	*T* = 296 (2) K
β = 96.882 (1)º	Block, colourless
γ = 103.542 (1)º	0.43 × 0.43 × 0.43 mm
*V* = 1157.5 (2) Å^3^	

### Data collection

**Table:**

Bruker SMART area-detector diffractometer	3669 reflections with *I* > 2σ(*I*)
Radiation source: fine-focus sealed tube	*R*_int_ = 0.019
Monochromator: graphite	θ_max_ = 27.5º
*T* = 296(2) K	θ_min_ = 2.5º
φ and ω scans	*h* = −12→13
Absorption correction: None	*k* = −14→14
10086 measured reflections	*l* = −15→15
5195 independent reflections	

### Refinement

**Table:**

Refinement on *F*^2^	Secondary atom site location: difference Fourier map
Least-squares matrix: full	Hydrogen site location: inferred from neighbouring sites
*R*[*F*^2^ > 2σ(*F*^2^)] = 0.046	H-atom parameters constrained
*wR*(*F*^2^) = 0.151	*w* = 1/[σ^2^(*F*_o_^2^) + (0.0768*P*)^2^ + 0.3784*P*] where *P* = (*F*_o_^2^ + 2*F*_c_^2^)/3
*S* = 1.03	(Δ/σ)_max_ = 0.001
5195 reflections	Δρ_max_ = 0.34 e Å^−^^3^
318 parameters	Δρ_min_ = −0.25 e Å^−^^3^
30 restraints	Extinction correction: none
Primary atom site location: structure-invariant direct methods	

### Fractional atomic coordinates and isotropic or equivalent isotropic displacement parameters (Å^2^)

**Table:**

	*x*	*y*	*z*	*U*_iso_*/*U*_eq_	Occ. (<1)
S1	0.43383 (7)	0.52869 (8)	0.19023 (8)	0.0736 (2)	
S2	0.86262 (7)	0.60297 (6)	0.65571 (5)	0.05353 (19)	
F1	0.89175 (19)	0.80884 (18)	0.17554 (17)	0.0805 (5)	
F2	0.8985 (2)	0.6035 (2)	0.06303 (14)	0.0868 (6)	
F3	1.16124 (19)	0.8540 (2)	0.22574 (18)	0.0943 (6)	
F4	1.1390 (2)	0.6431 (2)	0.19936 (18)	0.0921 (6)	
F5	1.10459 (17)	0.92568 (14)	0.44135 (15)	0.0707 (4)	
F6	1.19297 (14)	0.76341 (19)	0.43741 (16)	0.0735 (5)	
O1'	0.947 (3)	0.8292 (9)	0.9172 (7)	0.091 (4)	0.64 (5)
O1	1.010 (3)	0.8304 (18)	0.9249 (10)	0.080 (4)	0.36 (5)
C1	0.5164 (7)	0.8436 (7)	0.3633 (7)	0.104 (3)	0.788 (17)
H1A	0.5476	0.9370	0.4276	0.156*	0.788 (17)
H1B	0.4403	0.7889	0.3812	0.156*	0.788 (17)
H1C	0.4848	0.8435	0.2846	0.156*	0.788 (17)
C1'	0.577 (3)	0.8783 (13)	0.3189 (17)	0.077 (6)	0.212 (17)
H1'A	0.6029	0.9682	0.3880	0.116*	0.212 (17)
H1'B	0.4767	0.8414	0.2910	0.116*	0.212 (17)
H1'C	0.6169	0.8861	0.2522	0.116*	0.212 (17)
C2	0.6323 (4)	0.7854 (3)	0.3580 (3)	0.0885 (10)	
H2A	0.6646	0.7900	0.4393	0.106*	
H2B	0.7092	0.8446	0.3433	0.106*	
C3	0.6050 (3)	0.6395 (3)	0.2628 (2)	0.0565 (6)	
C4	0.7027 (2)	0.5769 (2)	0.2221 (2)	0.0454 (5)	
C5	0.6422 (3)	0.4353 (2)	0.1294 (2)	0.0519 (6)	
C6	0.7084 (3)	0.3380 (3)	0.0696 (2)	0.0677 (7)	
H6	0.8061	0.3627	0.0841	0.081*	
C7	0.6267 (5)	0.2048 (3)	−0.0111 (3)	0.0914 (11)	
H7	0.6703	0.1400	−0.0517	0.110*	
C8	0.4127 (4)	0.2581 (4)	0.0223 (3)	0.0846 (10)	
H8	0.3149	0.2315	0.0058	0.102*	
C9	0.4813 (5)	0.1652 (4)	−0.0332 (3)	0.0991 (13)	
H9	0.4292	0.0739	−0.0867	0.119*	
C10	0.4937 (3)	0.3945 (3)	0.1048 (2)	0.0615 (7)	
C11	0.8549 (2)	0.6521 (2)	0.2655 (2)	0.0424 (5)	
C12	0.9296 (3)	0.6987 (3)	0.1805 (2)	0.0560 (6)	
C13	1.0846 (3)	0.7499 (3)	0.2438 (2)	0.0576 (6)	
C14	1.0847 (2)	0.7868 (2)	0.3798 (2)	0.0480 (5)	
C15	0.9421 (2)	0.7024 (2)	0.37843 (19)	0.0387 (4)	
C16	0.9193 (2)	0.6924 (2)	0.49357 (19)	0.0385 (4)	
C17	0.9764 (2)	0.8081 (2)	0.6132 (2)	0.0467 (5)	
H17	1.0247	0.8966	0.6245	0.056*	
C18	0.9532 (3)	0.7762 (2)	0.7089 (2)	0.0533 (6)	
C19	0.9934 (4)	0.8673 (3)	0.8416 (3)	0.0801 (9)	
H19	1.0552	0.9562	0.8702	0.096*	0.64 (5)
H19'	1.0075	0.9613	0.8654	0.096*	0.36 (5)
C20	0.8536 (2)	0.5729 (2)	0.50312 (19)	0.0401 (4)	
C21	0.7831 (2)	0.4277 (2)	0.4049 (2)	0.0425 (5)	
H21A	0.8296	0.3668	0.4216	0.051*	
H21B	0.7949	0.4238	0.3247	0.051*	
C22	0.6266 (2)	0.3746 (2)	0.3974 (2)	0.0510 (5)	
H22A	0.6139	0.3879	0.4796	0.061*	
H22B	0.5782	0.4289	0.3713	0.061*	
C23	0.5601 (3)	0.2226 (3)	0.3076 (3)	0.0634 (7)	
H23A	0.6027	0.1675	0.3378	0.076*	
H23B	0.5800	0.2077	0.2271	0.076*	
C24	0.4014 (4)	0.1731 (4)	0.2918 (3)	0.0940 (11)	
H24A	0.3648	0.0766	0.2346	0.141*	
H24B	0.3583	0.2255	0.2598	0.141*	
H24C	0.3810	0.1860	0.3709	0.141*	

### Atomic displacement parameters (Å^2^)

**Table:**

	*U*^11^	*U*^22^	*U*^33^	*U*^12^	*U*^13^	*U*^23^
S1	0.0440 (4)	0.0835 (5)	0.0917 (6)	0.0164 (3)	−0.0006 (3)	0.0430 (4)
S2	0.0674 (4)	0.0499 (3)	0.0462 (3)	0.0127 (3)	0.0157 (3)	0.0262 (3)
F1	0.0839 (11)	0.0906 (12)	0.0950 (12)	0.0251 (9)	0.0131 (9)	0.0717 (11)
F2	0.0934 (13)	0.0972 (13)	0.0451 (9)	−0.0039 (10)	0.0150 (8)	0.0255 (9)
F3	0.0792 (12)	0.1137 (15)	0.0913 (13)	−0.0117 (10)	0.0169 (10)	0.0701 (12)
F4	0.0853 (12)	0.1121 (15)	0.0909 (13)	0.0522 (11)	0.0407 (10)	0.0382 (12)
F5	0.0776 (10)	0.0444 (8)	0.0744 (10)	−0.0017 (7)	0.0125 (8)	0.0233 (7)
F6	0.0408 (7)	0.1085 (13)	0.0919 (11)	0.0184 (8)	0.0082 (7)	0.0688 (11)
O1'	0.127 (8)	0.085 (3)	0.047 (2)	0.009 (4)	0.025 (3)	0.0263 (19)
O1	0.107 (9)	0.084 (5)	0.053 (4)	0.032 (5)	0.015 (4)	0.033 (3)
C1	0.098 (4)	0.102 (4)	0.114 (4)	0.061 (3)	0.027 (3)	0.031 (3)
C1'	0.082 (10)	0.072 (8)	0.084 (9)	0.033 (7)	0.013 (6)	0.036 (6)
C2	0.080 (2)	0.0667 (19)	0.102 (2)	0.0363 (16)	0.0055 (18)	0.0168 (18)
C3	0.0474 (13)	0.0579 (14)	0.0639 (15)	0.0171 (11)	0.0035 (11)	0.0285 (12)
C4	0.0462 (12)	0.0475 (12)	0.0416 (11)	0.0112 (9)	0.0022 (9)	0.0226 (10)
C5	0.0569 (14)	0.0512 (13)	0.0419 (12)	0.0071 (10)	0.0020 (10)	0.0228 (10)
C6	0.0835 (19)	0.0575 (15)	0.0530 (15)	0.0147 (14)	0.0191 (14)	0.0182 (12)
C7	0.128 (3)	0.0590 (18)	0.0636 (19)	0.0153 (19)	0.028 (2)	0.0098 (15)
C8	0.077 (2)	0.075 (2)	0.0695 (19)	−0.0144 (17)	−0.0182 (16)	0.0302 (17)
C9	0.126 (3)	0.062 (2)	0.063 (2)	−0.012 (2)	−0.001 (2)	0.0117 (16)
C10	0.0552 (14)	0.0627 (15)	0.0549 (14)	0.0001 (12)	−0.0070 (11)	0.0298 (12)
C11	0.0446 (11)	0.0412 (11)	0.0440 (11)	0.0122 (9)	0.0084 (9)	0.0225 (9)
C12	0.0625 (15)	0.0605 (14)	0.0468 (13)	0.0110 (12)	0.0099 (11)	0.0308 (12)
C13	0.0538 (14)	0.0631 (15)	0.0651 (16)	0.0132 (12)	0.0200 (12)	0.0381 (13)
C14	0.0416 (11)	0.0476 (12)	0.0573 (14)	0.0091 (9)	0.0057 (10)	0.0296 (11)
C15	0.0392 (10)	0.0366 (10)	0.0445 (11)	0.0130 (8)	0.0096 (8)	0.0213 (9)
C16	0.0366 (10)	0.0395 (10)	0.0430 (11)	0.0134 (8)	0.0057 (8)	0.0215 (9)
C17	0.0537 (12)	0.0384 (11)	0.0466 (12)	0.0122 (9)	0.0082 (10)	0.0192 (9)
C18	0.0645 (15)	0.0470 (12)	0.0453 (12)	0.0153 (11)	0.0112 (11)	0.0186 (10)
C19	0.126 (3)	0.0564 (16)	0.0457 (15)	0.0148 (17)	0.0188 (16)	0.0175 (13)
C20	0.0404 (10)	0.0419 (11)	0.0431 (11)	0.0151 (8)	0.0092 (9)	0.0224 (9)
C21	0.0455 (11)	0.0382 (10)	0.0476 (12)	0.0153 (9)	0.0087 (9)	0.0218 (9)
C22	0.0471 (12)	0.0458 (12)	0.0566 (14)	0.0088 (10)	0.0090 (10)	0.0229 (11)
C23	0.0630 (16)	0.0483 (13)	0.0684 (17)	0.0043 (12)	0.0049 (13)	0.0253 (12)
C24	0.073 (2)	0.079 (2)	0.091 (2)	−0.0168 (17)	0.0107 (17)	0.0238 (18)

### Geometric parameters (Å, °)

**Table:**

S1—C10	1.726 (3)	C7—H7	0.9300
S1—C3	1.742 (3)	C8—C9	1.369 (5)
S2—C20	1.717 (2)	C8—C10	1.403 (4)
S2—C18	1.725 (2)	C8—H8	0.9300
F1—C12	1.376 (3)	C9—H9	0.9300
F2—C12	1.331 (3)	C11—C15	1.346 (3)
F3—C13	1.335 (3)	C11—C12	1.512 (3)
F4—C13	1.360 (3)	C12—C13	1.513 (4)
F5—C14	1.366 (3)	C13—C14	1.521 (3)
F6—C14	1.350 (3)	C14—C15	1.510 (3)
O1'—C19	1.236 (5)	C15—C16	1.471 (3)
O1—C19	1.228 (8)	C16—C20	1.385 (3)
C1—C2	1.453 (5)	C16—C17	1.430 (3)
C1—H1A	0.9600	C17—C18	1.358 (3)
C1—H1B	0.9600	C17—H17	0.9300
C1—H1C	0.9600	C18—C19	1.453 (4)
C1'—C2	1.468 (8)	C19—H19	0.9300
C1'—H1'A	0.9600	C19—H19'	0.9300
C1'—H1'B	0.9600	C20—C21	1.497 (3)
C1'—H1'C	0.9600	C21—C22	1.523 (3)
C2—C3	1.499 (4)	C21—H21A	0.9700
C2—H2A	0.9700	C21—H21B	0.9700
C2—H2B	0.9700	C22—C23	1.518 (3)
C3—C4	1.359 (3)	C22—H22A	0.9700
C4—C5	1.441 (3)	C22—H22B	0.9700
C4—C11	1.475 (3)	C23—C24	1.522 (4)
C5—C6	1.399 (4)	C23—H23A	0.9700
C5—C10	1.412 (4)	C23—H23B	0.9700
C6—C7	1.378 (4)	C24—H24A	0.9600
C6—H6	0.9300	C24—H24B	0.9600
C7—C9	1.384 (6)	C24—H24C	0.9600
			
C10—S1—C3	91.85 (12)	F4—C13—C12	108.6 (2)
C20—S2—C18	92.17 (11)	F3—C13—C14	113.6 (2)
C2—C1—H1A	109.5	F4—C13—C14	108.9 (2)
C2—C1—H1B	109.5	C12—C13—C14	103.50 (19)
H1A—C1—H1B	109.5	F6—C14—F5	105.70 (19)
C2—C1—H1C	109.5	F6—C14—C15	113.35 (17)
H1A—C1—H1C	109.5	F5—C14—C15	111.64 (19)
H1B—C1—H1C	109.5	F6—C14—C13	111.9 (2)
C2—C1'—H1'A	109.5	F5—C14—C13	108.90 (18)
C2—C1'—H1'B	109.5	C15—C14—C13	105.39 (18)
H1'A—C1'—H1'B	109.5	C11—C15—C16	131.72 (19)
C2—C1'—H1'C	109.5	C11—C15—C14	109.62 (18)
H1'A—C1'—H1'C	109.5	C16—C15—C14	118.62 (18)
H1'B—C1'—H1'C	109.5	C20—C16—C17	111.60 (18)
C1—C2—C3	117.8 (3)	C20—C16—C15	126.15 (19)
C1'—C2—C3	117.9 (6)	C17—C16—C15	122.02 (18)
C1—C2—H2A	107.9	C18—C17—C16	113.4 (2)
C1'—C2—H2A	132.3	C18—C17—H17	123.3
C3—C2—H2A	107.9	C16—C17—H17	123.3
C1—C2—H2B	107.9	C17—C18—C19	128.5 (2)
C3—C2—H2B	107.9	C17—C18—S2	111.32 (18)
H2A—C2—H2B	107.2	C19—C18—S2	120.2 (2)
C4—C3—C2	127.0 (2)	O1—C19—C18	125.4 (8)
C4—C3—S1	111.98 (18)	O1'—C19—C18	121.5 (5)
C2—C3—S1	121.0 (2)	O1—C19—H19	107.4
C3—C4—C5	113.4 (2)	O1'—C19—H19	119.3
C3—C4—C11	121.9 (2)	C18—C19—H19	119.3
C5—C4—C11	124.6 (2)	O1—C19—H19'	117.3
C6—C5—C10	119.1 (2)	O1'—C19—H19'	112.4
C6—C5—C4	129.8 (2)	C18—C19—H19'	117.3
C10—C5—C4	111.1 (2)	C16—C20—C21	130.78 (19)
C7—C6—C5	119.0 (3)	C16—C20—S2	111.48 (16)
C7—C6—H6	120.5	C21—C20—S2	117.72 (15)
C5—C6—H6	120.5	C20—C21—C22	113.64 (18)
C6—C7—C9	121.5 (3)	C20—C21—H21A	108.8
C6—C7—H7	119.2	C22—C21—H21A	108.8
C9—C7—H7	119.2	C20—C21—H21B	108.8
C9—C8—C10	118.4 (3)	C22—C21—H21B	108.8
C9—C8—H8	120.8	H21A—C21—H21B	107.7
C10—C8—H8	120.8	C23—C22—C21	113.0 (2)
C8—C9—C7	121.1 (3)	C23—C22—H22A	109.0
C8—C9—H9	119.4	C21—C22—H22A	109.0
C7—C9—H9	119.4	C23—C22—H22B	109.0
C8—C10—C5	120.9 (3)	C21—C22—H22B	109.0
C8—C10—S1	127.5 (3)	H22A—C22—H22B	107.8
C5—C10—S1	111.61 (19)	C22—C23—C24	112.8 (2)
C15—C11—C4	130.17 (19)	C22—C23—H23A	109.0
C15—C11—C12	110.50 (19)	C24—C23—H23A	109.0
C4—C11—C12	118.92 (19)	C22—C23—H23B	109.0
F2—C12—F1	105.7 (2)	C24—C23—H23B	109.0
F2—C12—C11	114.8 (2)	H23A—C23—H23B	107.8
F1—C12—C11	109.4 (2)	C23—C24—H24A	109.5
F2—C12—C13	113.8 (2)	C23—C24—H24B	109.5
F1—C12—C13	108.2 (2)	H24A—C24—H24B	109.5
C11—C12—C13	104.97 (18)	C23—C24—H24C	109.5
F3—C13—F4	107.5 (2)	H24A—C24—H24C	109.5
F3—C13—C12	114.5 (2)	H24B—C24—H24C	109.5
			
C1—C2—C3—C4	163.3 (6)	C11—C12—C13—C14	−23.4 (2)
C1'—C2—C3—C4	119.3 (14)	F3—C13—C14—F6	−88.3 (3)
C1—C2—C3—S1	−16.7 (7)	F4—C13—C14—F6	31.4 (3)
C1'—C2—C3—S1	−60.7 (14)	C12—C13—C14—F6	146.9 (2)
C10—S1—C3—C4	0.8 (2)	F3—C13—C14—F5	28.1 (3)
C10—S1—C3—C2	−179.3 (3)	F4—C13—C14—F5	147.89 (19)
C2—C3—C4—C5	−179.9 (3)	C12—C13—C14—F5	−96.7 (2)
S1—C3—C4—C5	0.1 (3)	F3—C13—C14—C15	148.0 (2)
C2—C3—C4—C11	−3.0 (4)	F4—C13—C14—C15	−92.2 (2)
S1—C3—C4—C11	176.99 (17)	C12—C13—C14—C15	23.2 (2)
C3—C4—C5—C6	−177.8 (3)	C4—C11—C15—C16	4.6 (4)
C11—C4—C5—C6	5.4 (4)	C12—C11—C15—C16	177.0 (2)
C3—C4—C5—C10	−1.1 (3)	C4—C11—C15—C14	−172.9 (2)
C11—C4—C5—C10	−177.9 (2)	C12—C11—C15—C14	−0.5 (2)
C10—C5—C6—C7	−0.4 (4)	F6—C14—C15—C11	−137.4 (2)
C4—C5—C6—C7	176.1 (3)	F5—C14—C15—C11	103.4 (2)
C5—C6—C7—C9	−0.6 (5)	C13—C14—C15—C11	−14.7 (2)
C10—C8—C9—C7	−1.4 (5)	F6—C14—C15—C16	44.7 (3)
C6—C7—C9—C8	1.6 (6)	F5—C14—C15—C16	−74.5 (2)
C9—C8—C10—C5	0.3 (4)	C13—C14—C15—C16	167.42 (19)
C9—C8—C10—S1	−177.5 (3)	C11—C15—C16—C20	47.7 (3)
C6—C5—C10—C8	0.5 (4)	C14—C15—C16—C20	−135.0 (2)
C4—C5—C10—C8	−176.5 (2)	C11—C15—C16—C17	−138.3 (2)
C6—C5—C10—S1	178.73 (19)	C14—C15—C16—C17	39.1 (3)
C4—C5—C10—S1	1.7 (3)	C20—C16—C17—C18	−0.5 (3)
C3—S1—C10—C8	176.7 (3)	C15—C16—C17—C18	−175.3 (2)
C3—S1—C10—C5	−1.4 (2)	C16—C17—C18—C19	−178.8 (3)
C3—C4—C11—C15	68.7 (3)	C16—C17—C18—S2	0.8 (3)
C5—C4—C11—C15	−114.7 (3)	C20—S2—C18—C17	−0.7 (2)
C3—C4—C11—C12	−103.1 (3)	C20—S2—C18—C19	178.9 (3)
C5—C4—C11—C12	73.5 (3)	C17—C18—C19—O1	−156.0 (19)
C15—C11—C12—F2	141.2 (2)	S2—C18—C19—O1	25 (2)
C4—C11—C12—F2	−45.4 (3)	C17—C18—C19—O1'	168.9 (15)
C15—C11—C12—F1	−100.3 (2)	S2—C18—C19—O1'	−10.6 (16)
C4—C11—C12—F1	73.1 (3)	C17—C16—C20—C21	−178.2 (2)
C15—C11—C12—C13	15.6 (3)	C15—C16—C20—C21	−3.6 (3)
C4—C11—C12—C13	−171.1 (2)	C17—C16—C20—S2	−0.1 (2)
F2—C12—C13—F3	86.1 (3)	C15—C16—C20—S2	174.49 (16)
F1—C12—C13—F3	−30.9 (3)	C18—S2—C20—C16	0.43 (17)
C11—C12—C13—F3	−147.6 (2)	C18—S2—C20—C21	178.80 (17)
F2—C12—C13—F4	−34.0 (3)	C16—C20—C21—C22	−117.1 (2)
F1—C12—C13—F4	−151.1 (2)	S2—C20—C21—C22	64.9 (2)
C11—C12—C13—F4	92.2 (2)	C20—C21—C22—C23	−173.65 (19)
F2—C12—C13—C14	−149.7 (2)	C21—C22—C23—C24	−175.0 (2)
F1—C12—C13—C14	93.3 (2)		
